# Metal and metal-free photocatalysts: mechanistic approach and application as photoinitiators of photopolymerization

**DOI:** 10.3762/bjoc.10.83

**Published:** 2014-04-15

**Authors:** Jacques Lalevée, Sofia Telitel, Pu Xiao, Marc Lepeltier, Frédéric Dumur, Fabrice Morlet-Savary, Didier Gigmes, Jean-Pierre Fouassier

**Affiliations:** 1 Institut de Science des Matériaux de Mulhouse IS2M – UMR CNRS 7361 –UHA; 15 rue Jean Starcky, 68057 Mulhouse Cedex, France; 2 Institut Lavoisier de Versailles, UMR 8180 CNRS, Université de Versailles Saint-Quentin en Yvelines, 45 avenue des Etats-Unis, 78035 Versailles Cedex, France; 3 Aix-Marseille Université, CNRS, Institut de Chimie Radicalaire, UMR 7273, F-13397 Marseille, France; 4 Univ. Bordeaux, IMS, UMR 5218, F-33400 Talence, France; 5 CNRS, IMS, UMR 5218, F-33400 Talence, France; 6 formerly: University of Haute Alsace/ENSCMu, 3 rue Alfred Werner, 68093 Mulhouse Cedex, France

**Keywords:** LEDs, photoinitiators, photopolymerization, photoredox catalysis

## Abstract

In the present paper, the photoredox catalysis is presented as a unique approach in the field of photoinitiators of polymerization. The principal photocatalysts already reported as well as the typical oxidation and reduction agents used in both reductive or oxidative cycles are gathered. The chemical mechanisms associated with various systems are also given. As compared to classical iridium-based photocatalysts which are mainly active upon blue light irradiation, a new photocatalyst Ir(piq)_2_(tmd) (also known as bis(1-phenylisoquinolinato-*N*,*C*^2’^)iridium(2,2,6,6-tetramethyl-3,5-heptanedionate) is also proposed as an example of green light photocatalyst (toward the long wavelength irradiation). The chemical mechanisms associated with Ir(piq)_2_(tmd) are investigated by ESR spin-trapping, laser flash photolysis, steady state photolysis, cyclic voltammetry and luminescence experiments.

## Introduction

Photoredox catalysis is now well-known and largely used in organic synthesis, especially in the development of sustainable radical-mediated chemical processes under very soft irradiation conditions (e.g., household fluorescence or LED bulbs, halogen lamps, sunlight, Xe lamp), e.g., enantioselective alkylation, cycloaddition, etc. [1–14]. Ruthenium- and iridium-based organometallic complexes are mostly employed as radical sources: they exhibit an excellent visible-light absorption, long lived excited states and suitable redox potentials and they work through either an oxidation or a reduction cycle [1–12]. To avoid, however, the high cost, potential toxicity and limited availability of these structures, metal-free organic dye compounds (e.g., Eosin-Y, Nile Red, Alizarine Red S, perylene derivative or Rhodamine B etc.) were recently proposed for cooperative asymmetric organophotoredox catalysis [13–14].

**Scheme 1:**
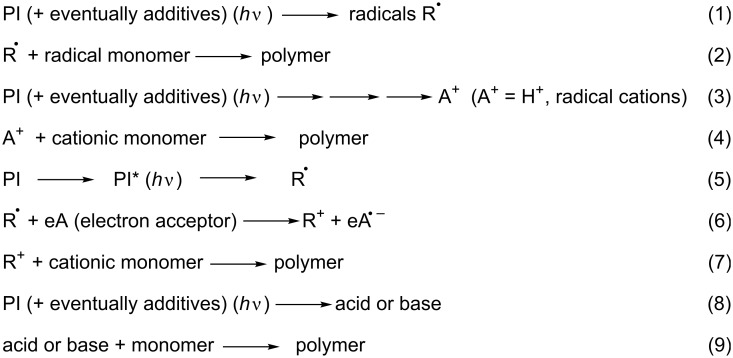
Examples of photoinitiating systems.

Photoredox catalysis was then introduced into the polymer photochemistry field (area) in the very past years (see a review in [15–22]). Indeed, in this area, free-radical photopolymerization (FRP, Scheme 1, reactions 1 and 2), cationic photopolymerization (CP, Scheme 1, reactions 3 and 4), free-radical promoted cationic photopolymerization (FRPCP, Scheme 1, reactions 5–7) or acid and base-catalyzed photocrosslinking (reactions 8 and 9) are initiated using photoinitiators (PI) which generate reactive species (radicals, cations, anions, radical cations, acids, bases). These PIs are often incorporated into multicomponent photoinitiating systems (PIS) where they primarily react with the other concomitant reagents or additives.

PIs and PISs have been extensively developed both in industrial R&D and academic laboratories [15–40]. PIs are usually organic molecules that are consumed during the light exposure [15–40]. The use of organometallic compounds as PIs was reported many years ago (see a review in [41]) and reintroduced in some recent papers dealing with, e.g., Cr, Ti, Fe, Rh, W, Pt, Ru, Ir, Zn, Zr-based derivatives [42–44]. On this occasion, it appeared that a photoredox catalysis behavior (allowing a PI regeneration while keeping a high reactivity/efficiency) can be introduced through a suitable selection of the PIs and PISs [45–55].

This approach opened up [45–53] a new way for the design of a novel high performance class of PIs for FRP and FRPCP (where the photoinitiator is now referred as a photoinitiator catalyst PIC). It brings, among others, the following novel properties [45–55]:

- Almost no photoinitiator is consumed.
- Since the spectral photosensitivity extends now from the UV to the visible, laser excitation in the purple, blue, yellow, green or red is feasible.
- Low light intensities (as delivered, e.g., by household lamps and LED bulbs) can be used; this is a catalytic process without loss of efficiency with irradiation.
- Photopolymerization under sunlight becomes reachable.
- The production of the radical or cationic initiating species for the FRP of acrylates or the FRPCP of epoxides, respectively, is quite easy; polymerization of sustainable monomers can also be achieved (e.g., epoxidized soybean oil).
- A possible dual behavior (simultaneous generation of radicals and cations that ensure the formation of, e.g., an epoxy/acrylate interpenetrated network IPN) is achieved.

**Figure 1:**
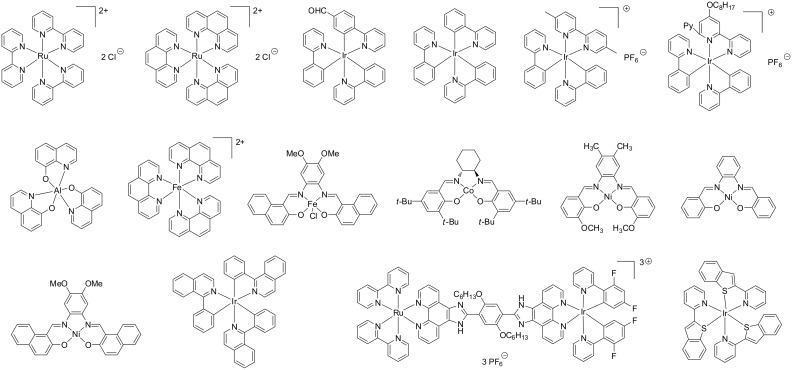
Previously reported PIC (based on metal complexes) [45–52].

**Figure 2:**
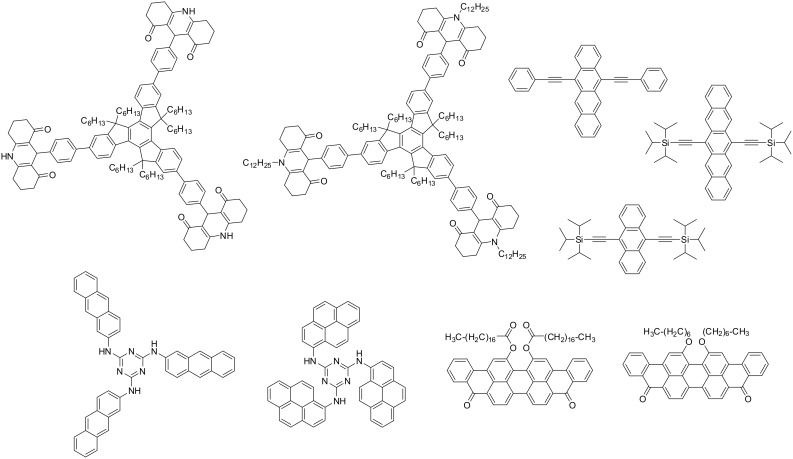
Previously reported PIC (metal free organic molecules) [54–55].

**Figure 3:**
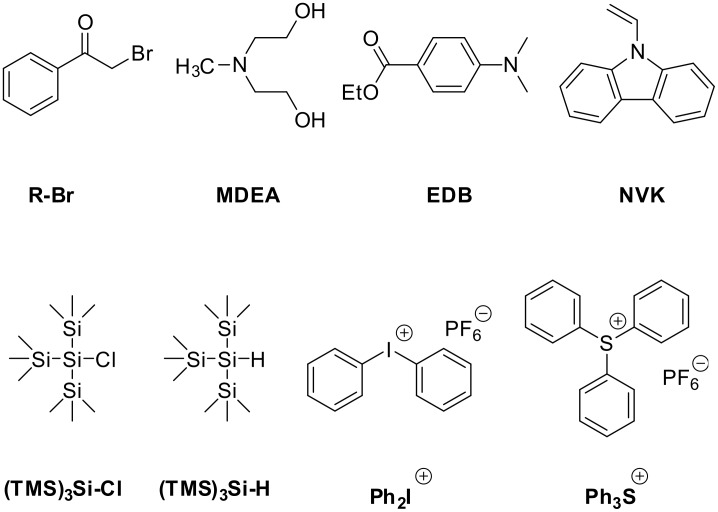
Typical oxidation and reduction agents used through the photoredox catalysis approach in polymerization photoinitiating systems.

**Figure 4:**
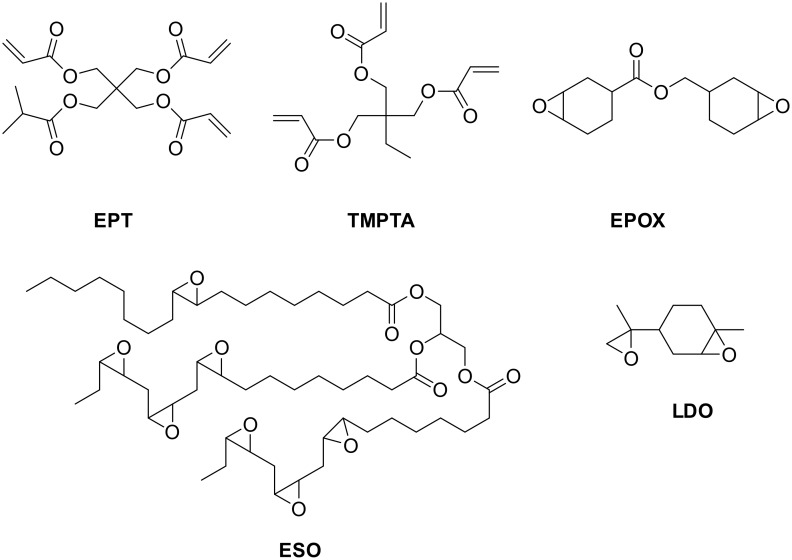
Typical monomers that can be polymerized through a photoredox catalysis approach.

Examples of PICs proposed by us in the photopolymerization reactions are depicted in Figure 1 and Figure 2 (for metal based PICs and metal free PICs, respectively) [45–55]. Their reactivity parameters (redox potentials, excited state lifetimes) are given in the associated references. These PICs are typically used (see below) in combination with various additives (see Figure 3 below) in three-component photoinitiating systems, e.g., based PIC/iodonium salt (or sulfonium salt)/tris(trimethylsilyl)silane (or *N*-vinylcarbazole) or PIC/amine/alkyl halide. Also, relatively high intensity light sources (Hg, Xe or Hg–Xe lamps, laser diodes) can be obviously employed. However, and with more interest, very soft irradiation conditions (using, e.g., household fluorescence or LED bulbs, halogen lamps or even sunlight) are also suitable to polymerize radical and cationic monomers (see Figure 4 below) under polychromatic or monochromatic lights in the 350–700 nm and to get tack free coatings.

The present paper will i) review the various possible mechanistic schemes using metal and metal free oxidizable or reducible PICs for FRP, CP and FRPCP, ii) show examples of reported PICs, iii) discuss the encountered mechanisms in various PIC based photoinitiating systems, iv) highlight some examples of high performance PISs and v) present the efficiency of a novel PIC in photopolymerization reactions as well as the excited state processes involved in the photoinitiating systems used.

## Experimental

**i) Compounds:** The synthesis of bis(1-phenylisoquinolinato-*N*,*C*^2’^)iridium(2,2,6,6-tetramethyl-3,5-heptanedionate) is described below (following a published procedure). All reagents and solvents were purchased from Aldrich or Alfa Aesar and used as received without further purification.

**ii) Irradiation sources:** Several lights were used: 1) polychromatic light from a halogen lamp (Fiber-Lite, DC-950 – incident light intensity: *I*_0_ ≈ 12 mW/cm^2^; in the 370–800 nm range); 2) monochromatic light delivered by a laser diode at 532 nm (*I*_0_ ≈ 100 mW/cm^2^) and 3) LEDs at 514 nm.

**iii) Free radical photopolymerization (FRP) of acrylates:** The experiments were carried out in laminated conditions or under air. The prepared formulations deposited on a BaF_2_ pellet (25 µm thick) were irradiated (see the irradiation devices). The evolution of the double bond content was continuously followed by real time FTIR spectroscopy (JASCO FTIR 4100) at about 1630 cm^−1^ as in [15].

**iv) The ring opening polymerization of epoxides:** The photosensitive formulations were deposited (25 µm thick) on a BaF_2_ pellet under air. The evolution of the epoxy group content was continuously followed by real time FTIR spectroscopy (JASCO FTIR 4100) at about 790 cm^−1^, respectively [15].

**v) ESR spin trapping (ESR-ST) experiments:** The ESR-ST experiments were carried out using an X-band spectrometer (MS 400 Magnettech). The radicals were produced at room temperature upon a light exposure (see the irradiation devices) under N_2_ and trapped by phenyl-*N-tert-*butylnitrone (PBN) according to a procedure described in detail in [15]. The ESR spectra simulations were carried out with the PEST WINSIM program.

## Results and Discussion

### Possible usable strategies for the design of PICs in the photopolymerization area

#### Oxidizable photoinitiator catalysts

**Scheme 2:**
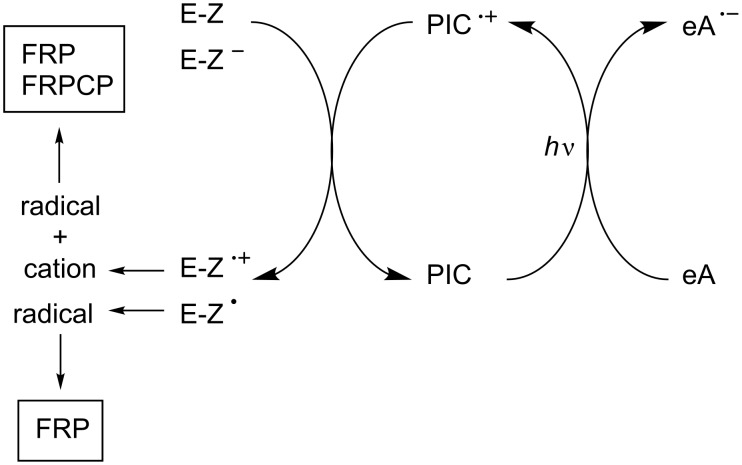
Reaction mechanisms for the three-component system PIC/eA/E-Z.

Using oxidizable photoinitiator catalysts, three possibilities can be briefly considered. The simplest first situation is depicted in Scheme 2. Through light excitation in the presence of an electron acceptor (eA), the oxidized form of the photoinitiator catalyst (PIC^•+^) is produced. A E-Z compound should be added to recover the PIC in its ground state and generate a radical and a cation. Suitable E-Z or E-Z^−^ structures have to be designed.

**Scheme 3:**
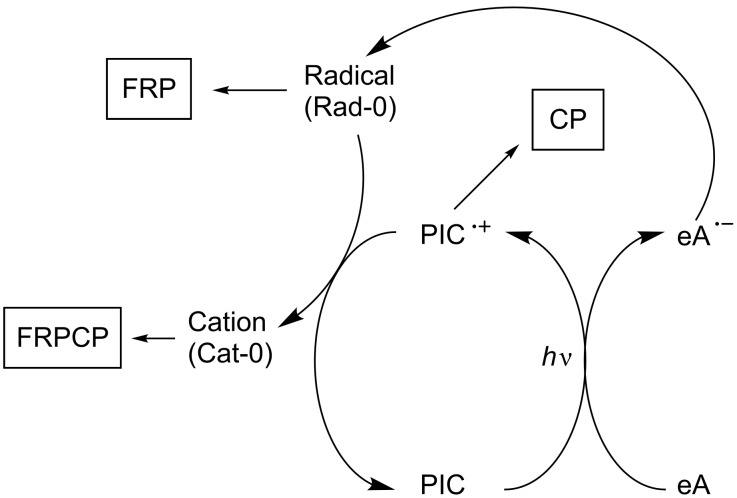
Reaction mechanisms for the two-component system PIC/eA.

A second situation is shown in Scheme 3. It consists in using the radical formed from the eA radical anion to recover the PIC. Both a radical and a cation are thus generated. The easiness of the radical → cation step as well as the nature of the cationic centers remains connected with the nature of eA. The key point is to find a radical directly formed through this way and that can be easily oxidized by PIC^•+^.

**Scheme 4:**
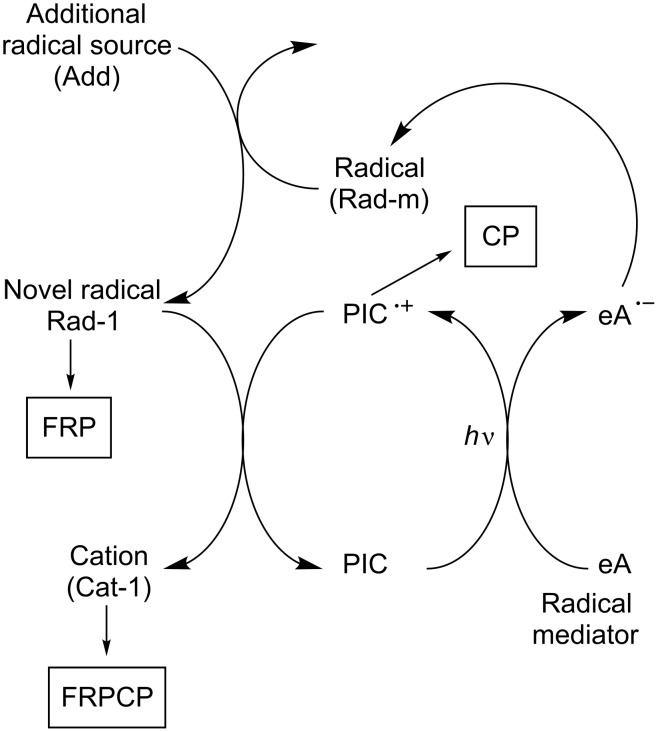
Reaction mechanisms for the system PIC/eA/add.

Another situation is encountered in Scheme 4 where the initially formed radical Rad-m (from the decomposition of a suitable electron donor as before) is converted into another radical Rad-1 (upon addition of a convenient radical source), this novel radical being able to favorably react with PIC^•+^. Such a process should be likely more feasible.

According to these different Schemes, both FRP, CP and FRPCP can be initiated from the free radicals and cations generated. In these three situations, as a function of its structure, the PIC radical cation PIC^•+^ can behave as an initiating species.

#### Reductible photoinitiator catalysts

**Scheme 5:**
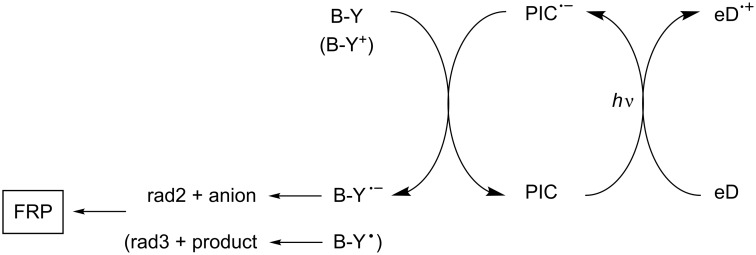
Reaction mechanisms for the system PIC/eD/B-Y.

Scheme 5 shows a situation where the PIC is reduced through a photoinduced electron transfer with an electron donor eD. A suitable B-Y (or B-Y^+^) compound leads to a regeneration of PIC and the formation of a radical and an anion (or a radical and a neutral product). Therefore, FRP can be achieved.

#### Examples of PICs in the photopolymerization area

Examples of PICs are shown above in Figure 1 and Figure 2. Typical oxidation and reduction agents used in catalytic (oxidation and reduction) cycles are gathered in Figure 3; the monomers that have been polymerized in previous works are given in Figure 4 [45–55].

#### Oxidizable photoinitiator catalysts

The oxidation of a PIC is quite easily realized. For example, the excitation of Ru(bpy)_3_^2+^ (with bpy = bipyridine) in the presence of an iodonium salt (e.g., Ph_2_I^+^) as eA leads to Ru(bpy)_3_^3+^. However, very few systems involving a E-Z structure or a E-Z anion (as suggested in Scheme 2) and allowing a regeneration of a ground state PIC together with efficient radical and cationic initiating species for FRP/CP/FRPCP has been reported yet [45–55].

Interestingly, when using a Ru complex as PIC and Ph_2_I^+^ salt as eA as above, a phenyl radical Ph^•^ is formed (Scheme 3). Unfortunately, the oxidation reaction of Ph^•^ by PIC^•+^ is rather hard [45–52] and such a system does not work. Sulfonium salts were also used as eA but the reactivity is lower than that for iodonium salts (see below) [56].

**Scheme 6:**
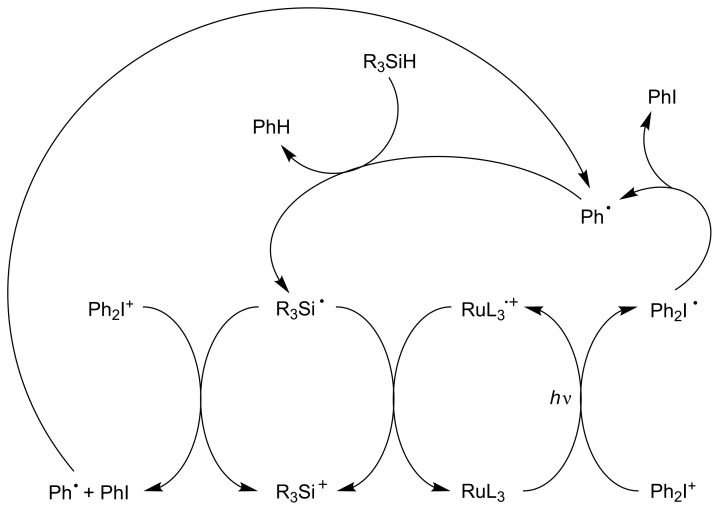
Reaction mechanisms for the system Ru(bpy)_3_^2+^/Ph_2_I^+^/R_3_SiH.

A typical efficient system based on Scheme 4 involving Ru(bpy)_3_^2+^ as PIC, Ph_2_I^+^ as eA and a silane R_3_SiH (e.g., tris(trimethylsilyl)silane TTMSS) as Add is detailed in Scheme 6. A phenyl radical is generated (previously noted Rad-m in Scheme 4). A silyl radical R_3_Si^•^ (noted Rad-1 above) and a silylium R_3_Si^+^ are formed through a subsequent Ph^•^/R_3_SiH hydrogen abstraction and R_3_Si^•^/PIC^•+^ interaction, respectively (the eA serves both as an electron donor and a radical mediator source) [45–52]. The Ph_2_I^+^/R_3_Si^•^ interaction increases the yields in both phenyl radicals and silylium species [45–52]. The nature of the PIC is responsible for the absorption properties. Interestingly, whatever the PIC, the nature of the cation is only dependent of the choice of Add. The three-component system behaves here as an efficient dual radical/cation source. Moreover, as already known [57], the introduction of the silane also reduces the oxygen inhibition of the radical stages of the FRP and FRPCP reactions.

Many photoinitiating systems have been designed on the basis of Scheme 6 [45–52]. Ruthenium and iridium-containing PICs are relatively well known and a large variety of derivatives have been recently tested in the photopolymerization area. Thus, more or less successful attempts using Fe, Pt, Ni, Zn-based complexes have been also recently reported [56,58–60].

**Scheme 7:**
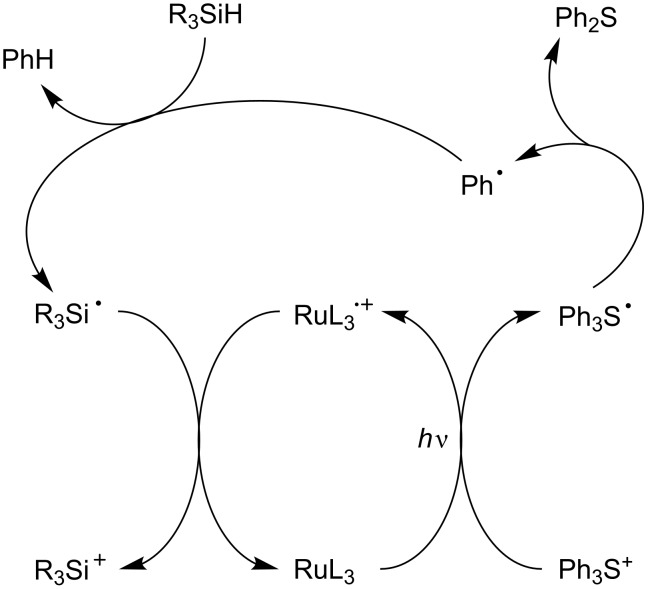
Reaction mechanisms for the Ru(ligand)_3_^2+^/Ph_3_S^+^/R_3_SiH system.

Other examples of eA and Add are also available. In some cases, a sulfonium salt (e.g., a triphenylsulfonium salt) can be introduced instead of the iodonium salt (Scheme 7) [56].

**Scheme 8:**
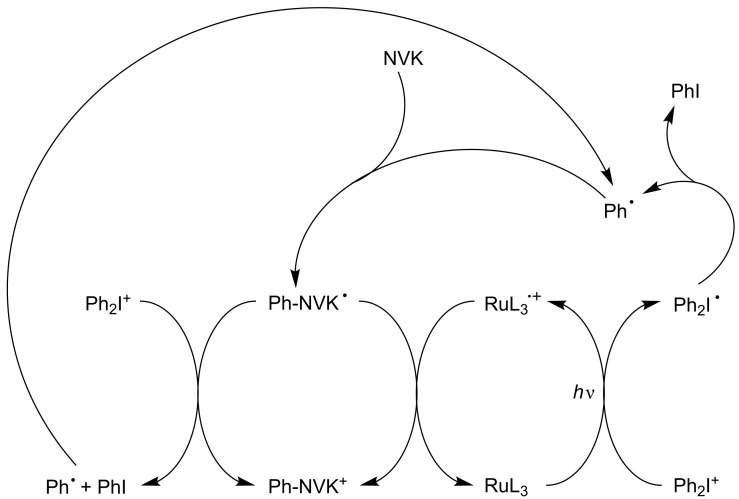
Reaction mechanisms for the Ru(ligand)_3_^2+^/Ph_2_I^+^/NVK system upon visible lights.

The silane has been also changed for *N*-vinylcarbazole NVK (Scheme 8). The phenyl radical adds to the NVK double bond and the resulting radical is electron rich and can be easily oxidized. NVK is a cheaper additive than silane (R_3_SiH) and exhibits a relatively similar performance in photoinitiating systems of cationic polymerization [45–52,54–55].

**Scheme 9:**
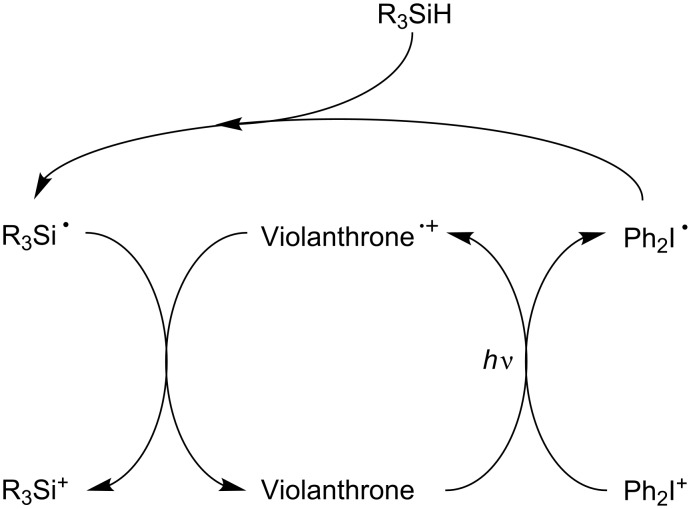
Reaction mechanisms for the violanthrone/Ph_2_I^+^/TTMSS (R_3_SiH) system upon red lights.

Examples of metal-free pure organic PICs for FRP and FRPCP have also been very recently reported [54–55,61–62]. For example, they involve a violanthrone dye Vi [61–62] or an anthracene derivative (e.g. bis[(triisopropylsilyl)ethynyl]anthracene) [54–55] as PIC, Ph_2_I^+^ as eA and TTMSS as Add (see the simplified Scheme 9 based on Scheme 6). Using violanthrone-79/Ph_2_I^+^/TTMSS allowed, for the first time, the formation of an initiating cationic species under a red laser line exposure at 635 nm. This result was very important as cationic polymerization in these irradiation conditions was not possible previously. Changing Vi or the anthracene derivative for a hydrocarbon (e.g. pyrene, naphthacene, pentacene) allows a tunable absorption of the system from 400 nm to 650 nm: exposure of the hydrocarbon/Ph_2_I^+^/TTMSS system to soft purple (405 nm), blue (457, 462 or 473 nm), green (514, 532 nm), yellow (591 nm) or red (630, 635 nm) LED bulbs or laser diodes becomes successful.

**Scheme 10:**
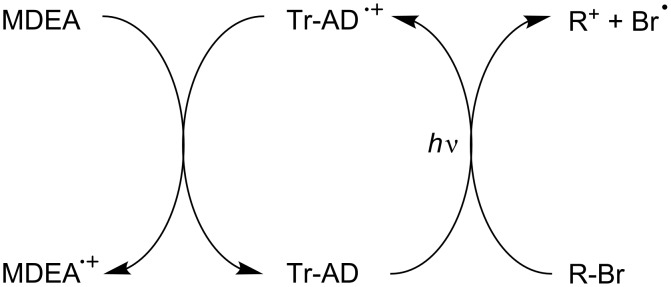
Reaction mechanisms for the Tr-AD/R-Br/MDEA system upon visible lights.

In rare examples, the PIC/methyldiethanolamine MDEA/phenacyl bromide R-Br (that usually operates through a reductive cycle; see below) works according to an oxidative cycle (Scheme 10). This is the case when the PIC stands for a truxene-acridinedione derivative Tr-AD (this is one of the possible examples for Scheme 2) [63]. For these PICs, the interaction of the excited states of Tr-AD with R-Br is much more favorable than the interaction with the amine.

**Scheme 11:**
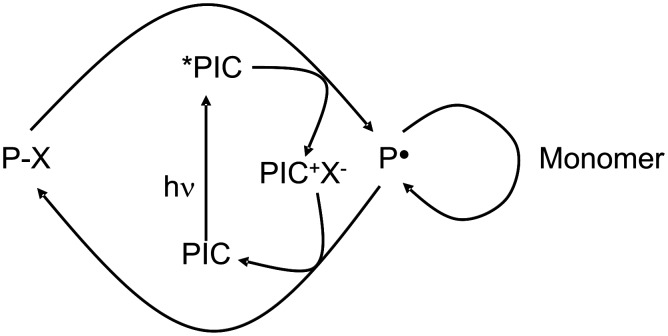
The photoredox catalysis for controlled polymerization reactions.

Recently, an iridium complex (Ir(ppy)_3_) (with ppy = 2-phenylpyridine) was proposed for the controlled photopolymerization reactions through a photoATRP process based on a photoredox catalysis approach (oxidative cycle; Scheme 11) [64].

All these described systems, producing radicals, cations or radical cations, allow efficient CP and FRPCP of cationic monomers, FRP of acrylates, simultaneous radical/cationic polymerization of epoxide/acrylate blend. The reactions can be carried out (see in [45–55]) in formulations containing multifunctional synthetic epoxides, acrylates, monomers/oligomers or epoxide/acrylate blends (renewable raw or modified materials are usable to some extent) with lights extending from the UV to the red, using polychromatic or monochromatic light sources, even with low intensity of light emission (sun, household devices).

#### Reductible photoinitiator catalysts

**Scheme 12:**
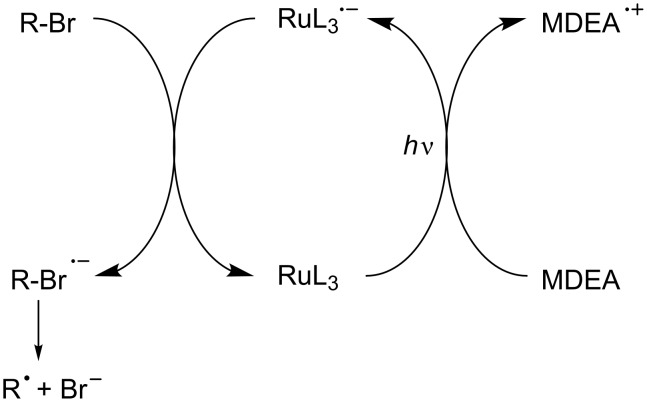
Reaction mechanisms for the Ru(ligand)_3_^2+^/MDEA/R-Br system upon visible lights.

Reported systems based on a reductive cycle are mainly based on Ru complexes [45–53]. Such a reductible PIC was first mentioned in FRP (Scheme 12) in a system composed of a Ru complex as PIC, an amine (methyldiethanolamine, MDEA) as an electron/proton donor and a phenacyl bromide R-Br [45]. In this mechanism, a reduced form of the Ru complex is formed (e.g., Ru(bpy)_3_^+^) and a phenacyl radical is produced upon the subsequent cleavage of the phenacyl halide radical anion. Later on, other amines (*N*,*N*-diisopropylethylamine, *N*,*N*-dimethylformamide) and bromides (ethyl 2-bromoisobutyrate, benzyl bromide) were proposed for the photocatalytic radical polymerization of various methacrylates [53]. Ir complexes can also be used.

As only carbon centered radicals can be generated in Scheme 12, the change of the phenacyl bromide for a compound being able to generate more efficient radicals towards the addition to double bonds was tentatively achieved in the Ru complex/amine (ethyl diaminoethylbenzoate)/chlorosilane (e.g., (TMS)_3_SiCl) system (see Scheme 12 and change R-Br and MDEA for (TMS)_3_SiCl and EDB, respectively); the performance in FRP remains unfortunately rather low [56]. Despite the presence of aminoalkyl and silyl initiating radicals (known as very reactive radicals towards the addition process onto acrylate double bond [65]), a lower reactivity of (TMS)_3_SiCl towards PIC^•−^ or/and a lower ability of the (TMS)_3_SiCl^•−^ radical anion to decompose into a silyl radical and a chlorine anion likely play a detrimental role. Bromosilanes might be considered but they are not easily accessible.

Works using metal-free PIC proceeding through a reduction cycle have been recently achieved in FRP for the first time [54–55]: they involve hydrocarbon derivatives (e.g., pyrene, naphtacene, pentacene), an amine (e.g., ethyl dimethylaminobenzoate) and an alkyl halide (e.g., phenacyl bromide); the mechanism is similar to that shown in Scheme 12. As above, a tunable absorption of the system is achieved by carefully selecting the hydrocarbon (the system is sensitive to lights ranging from 300 to 670 nm) [54–55]. Other colored PICs might be dyes (as suggested in experiments for organic synthesis purposes [54–56]) but preliminary experiments using common dyes such as Eosin suggest that their behavior as PICs is not straightforward. The redox processes with organic dyes are not always fully reversible: this plays a detrimental role in the catalytic cycle with a lost of the efficiency through a PIC degradation.

**Scheme 13:**
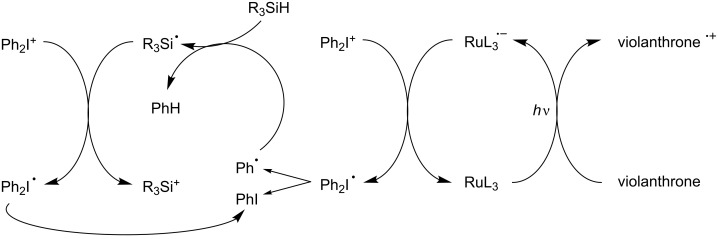
Reaction mechanisms for the Violanthrone/Ru(ligand)_3_^2+^/Ph_2_I^+^/R_3_SiH system upon visible lights.

A striking example concerns the use of a Ru complex (Ru(bpy)_3_^2+^) as PIC which is reduced here into Ru(bpy)_3_^+^ although as shown above, it usually works through an oxidation cycle [61–62]. A violanthrone derivative (violanthrone-79) is employed as eD and Ph_2_I^+^ as B-Y^+^ (see Scheme 5); TTMSS is added. This multicomponent system works according to the detailed Scheme 12. A phenyl radical is produced from B-Y^•^ (= Ph_2_I^•^). As shown before, the silane ensures the formation of R_3_Si^•^ and R_3_Si^+^ (Scheme 13), respectively [45–52]. This is presumably the most famous example so far for the generation of an initiating cation under a green laser line at 532 nm; the photosensitivity is extremely high.

**Scheme 14:**
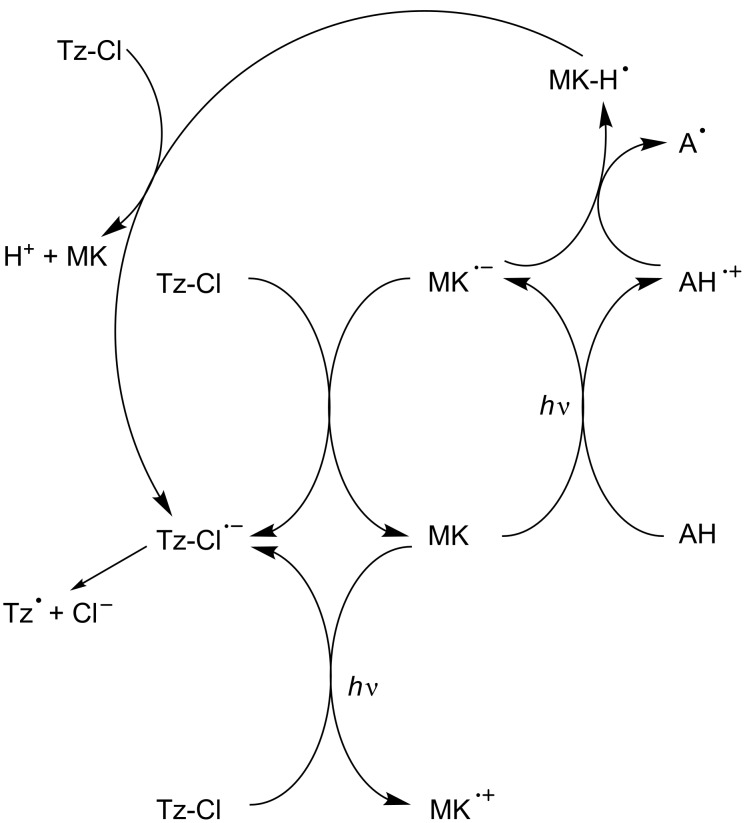
Reaction mechanisms for the MK/amine/triazine system upon visible lights.

A more complicated situation has also been found in some PIC/amine/alkyl halide system where both a reduction and an oxidation cycle simultaneously operate, e.g., in Michler’s ketone derivative MK/amine AH (e.g., MDEA)/chlorotriazine Tz-Cl (Scheme 14) [66].

All these reactions allowed the radical photopolymerization of formulations containing multifunctional synthetic acrylates using UV to red lights delivered by low intensity sources.

#### A new PIC based on a panchromatic iridium complex and the associated performance in the photopolymerization area

**Figure 5:**
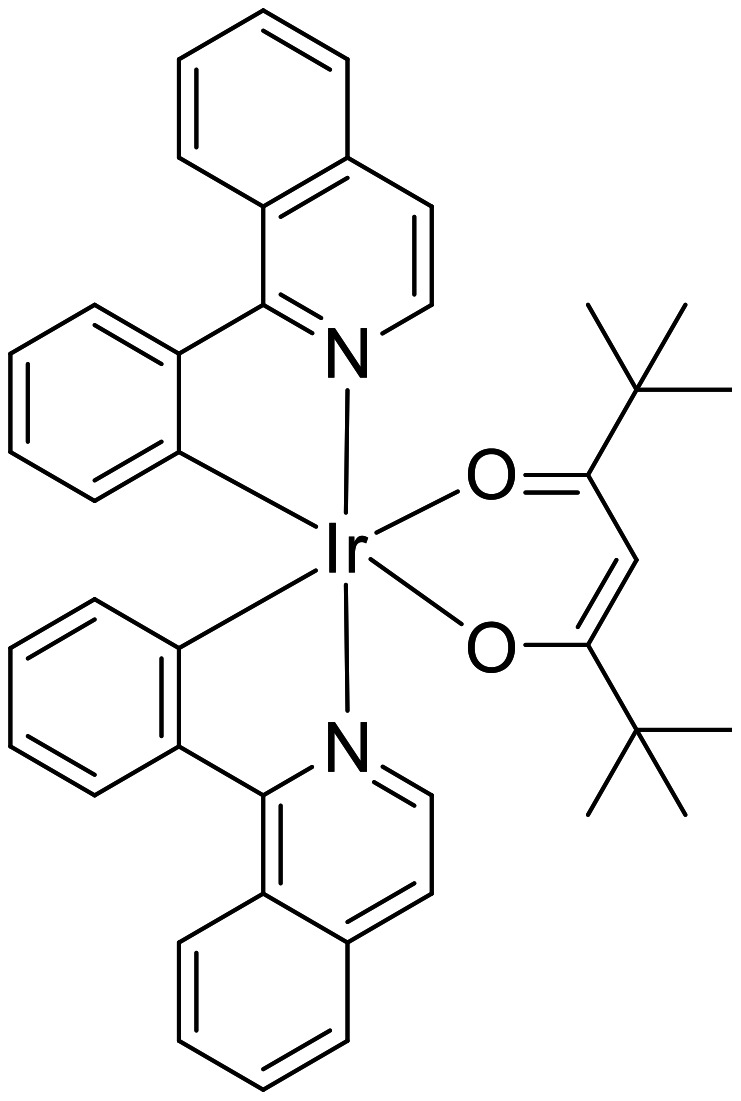
The new proposed PIC (Ir(piq)_2_(tmd)).

There is still a need for the development of new PICs with i) improved light absorption properties and ii) high reactivity. These new PICs can be highly worthwhile for the use of very soft irradiation conditions. For example, the actual Ir and Ru-based PICs are mainly characterized by a blue light absorption and are operative in the 400–480 nm spectral range, i.e., the well-known and commercially available Ir derivative Ir(ppy)_3_ (tris[2-phenylpyridinato-*C*^2^,*N*]iridium(III)) is characterized by an intense absorption at 370 nm and exhibits an only moderate absorption at λ > 400 nm. Among others, the design of green light sensitive Ir complexes is an interesting challenge. This is the reason why the new Ir based PIC (bis(1-phenylisoquinolinato-*N*,*C*^2’^)iridium(2,2,6,6-tetramethyl-3,5-heptanedionate) noted Ir(piq)_2_(tmd); Figure 5), already synthesized in [67] but never used as PIC in photoinitiating systems is presented here.

**Figure 6:**
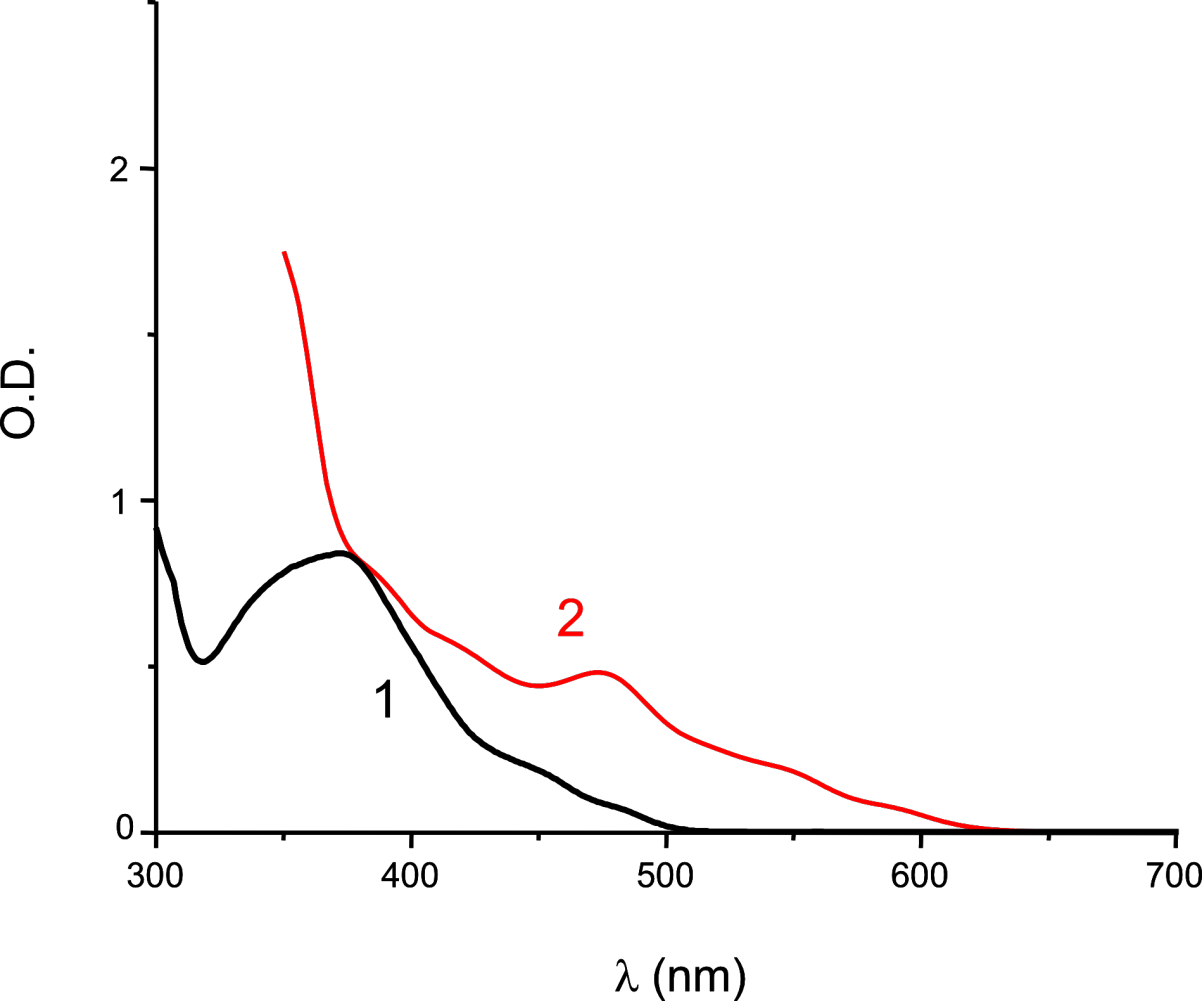
UV–visible light absorption spectra for Ir(piq)_2_(tmd) (2) and Ir(ppy)_3_ (1); solvent: acetonitrile.

Indeed, the visible light absorption properties of Ir(piq)_2_(tmd) are much better than those of Ir(ppy)_3_ (Figure 6). Remarkably, Ir(piq)_2_(tmd) is perfectly adapted for applications under green light exposure (LED bulb at 514 nm or a laser diode at 532 nm) and exhibits an almost panchromatic behavior in the 400–650 nm range.

### The PIC/Ph_2_I^+^/NVK system

#### The photochemical properties of Ir(piq)_2_(tmd)

A triplet-state lifetime of 1.1 μs has been determined for Ir(piq)_2_(tmd) by laser flash photolysis experiments. A relatively similar lifetime (1.3 μs) was previously measured for ^3^Ir(ppy)_3_ [68]. Such long lifetimes for triplet states are important in photoredox catalysis to ensure an efficient quenching by the oxidation agents.

**Figure 7:**
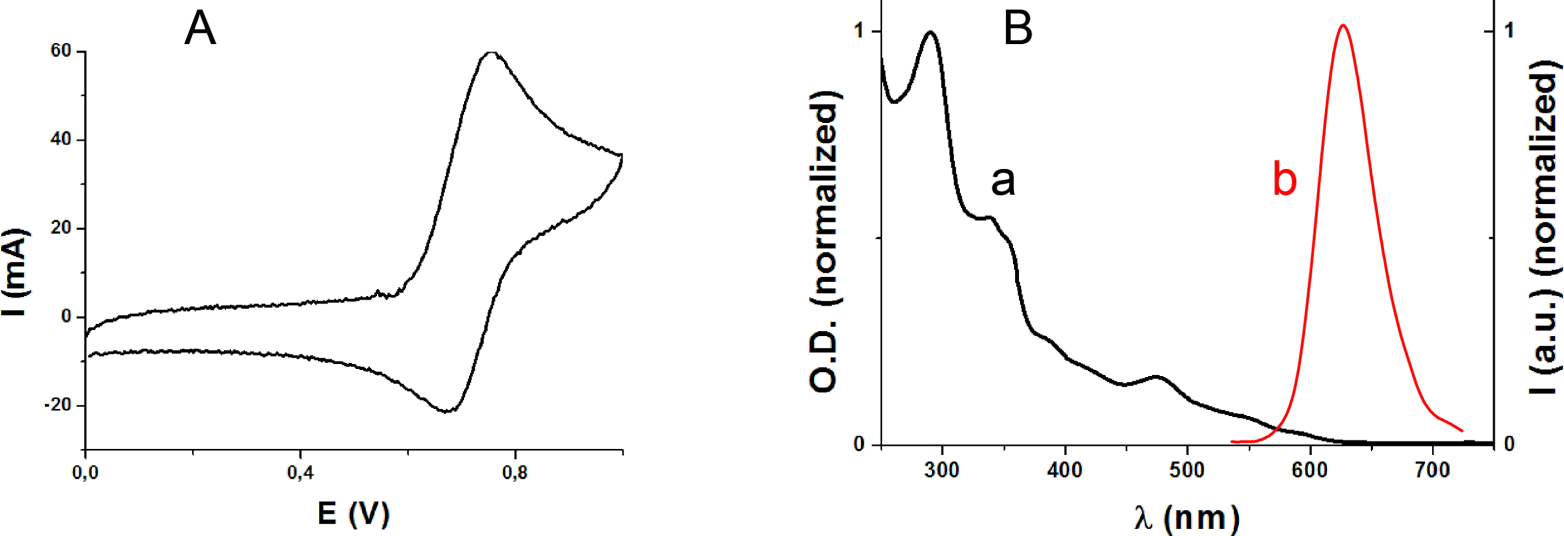
(A) cyclic voltamogramm for Ir(piq)_2_(tmd) in acetonitrile; (B) absorption (a) and luminescence (b) spectra for Ir(piq)_2_(tmd) (in acetonitrile).

Ir(piq)_2_(tmd) is characterized by an oxidation potential of 0.67 V vs SCE (Figure 7A); its triplet-state energy level (*E*_T_ ~ 2.07 eV) was determined from absorption/luminescence experiments (Figure 7B). From these data, a favorable ^3^Ir(piq)_2_(tmd)/Ph_2_I^+^ oxidation process can be expected, i.e., the free energy change Δ*G* for the corresponding electron transfer is negative (Δ*G* = −1.2 eV as calculated from the classical Rehm–Weller Δ*G* = *E*_ox_ – *E*_red_ – *E*_T_ + *C* where *E*_ox_, *E*_red_, *E*_T_ and *C* are the oxidation potential of Ir(piq)_2_(tmd), the reduction potential of Ph_2_I^+^, the excited triplet state energy of Ir(piq)_2_(tmd), and the electrostatic interaction energy for the initially formed ion pair, generally considered as negligible in polar solvents) [69].

**Figure 8:**
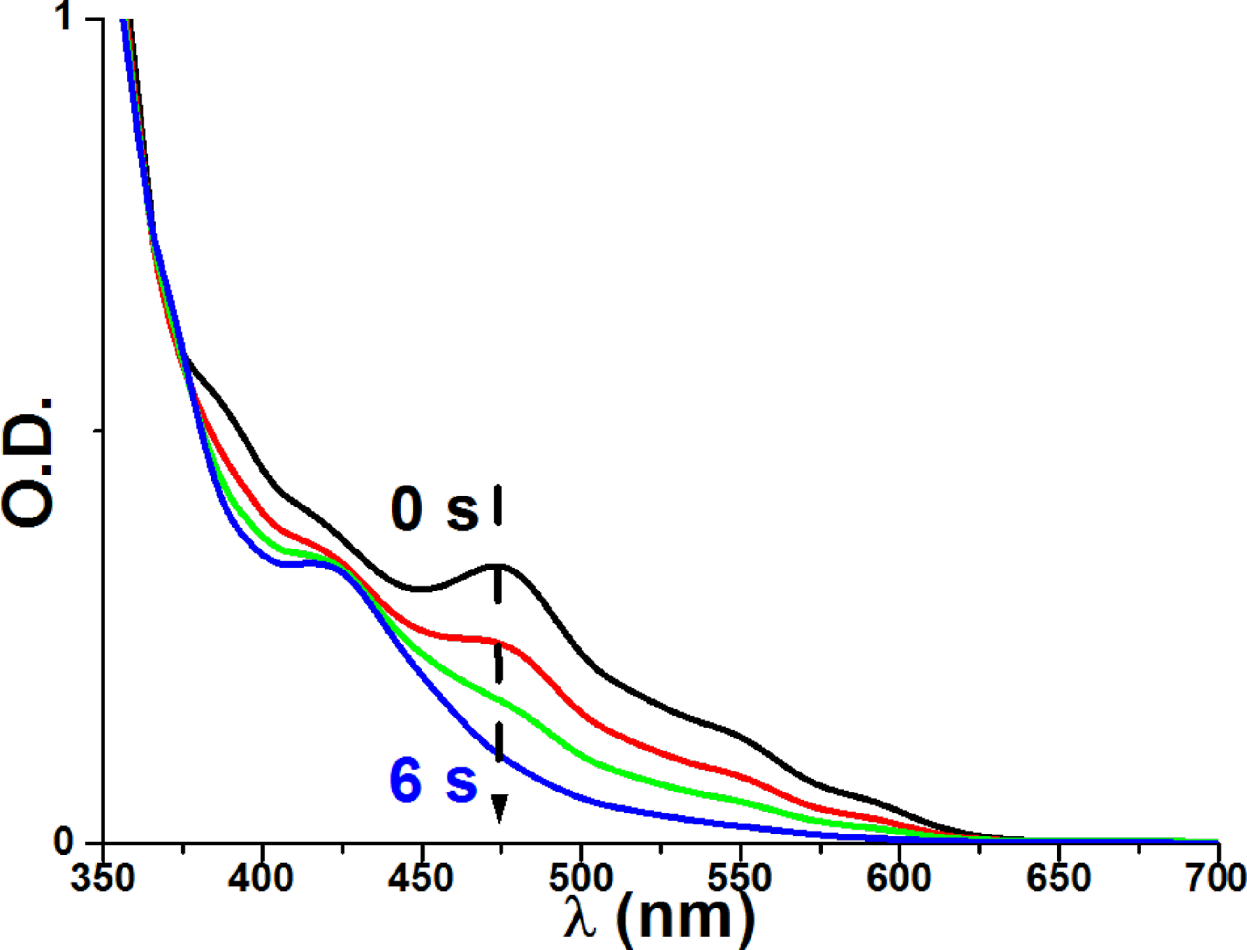
Photolysis of a Ir(piq)_2_(tmd)/Ph_2_I^+^ solution ([Ph_2_I^+^] = 0.023 M, in acetonitrile) upon a halogen lamp exposure.

**Figure 9:**
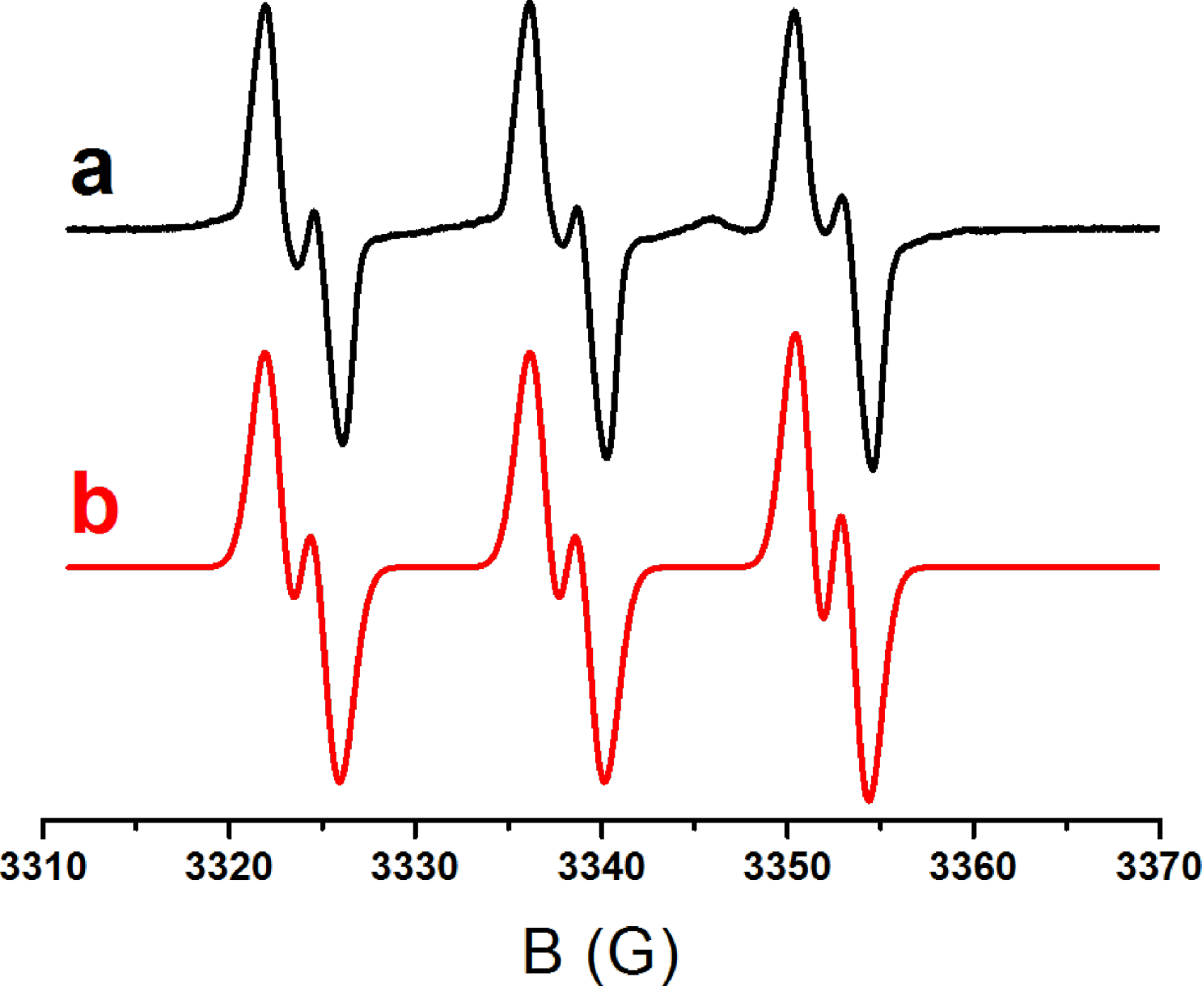
ESR spin-trapping spectra for the irradiation of a Ir(piq)_2_(tmd)/Ph_2_I^+^ solution in the presence of PBN; green LED bulb at 514 nm irradiation (the phenyl radical adducts are characterized by a_N_ = 14.2 G, a_H_ = 2.2 G, in full agreement with previous data) [45–52].

This favorable ^3^PIC/Ph_2_I^+^ interaction is also in line with a fast photolysis of the PIC observed during the irradiation of Ir(piq)_2_(tmd)/Ph_2_I^+^ (Figure 8). In ESR spin-trapping experiments on irradiated Ir(piq)_2_(tmd)/Ph_2_I^+^ in the presence of phenyl-*N*-*tert*-butylnitrone (PBN), phenyl radicals are also detected (Figure 9). In the presence of NVK, the formation of Ph-NVK^·^ is also observed (the PBN radical adducts are characterized by a_N_ = 14.4 G and a_H_ = 2.5 G, in agreement with previous data [45–52]). All these results are fully consistent with the Scheme 8 presented above. Therefore, Ir(piq)_2_(tmd) can be used in an oxidative cycle in combination with Ph_2_I^+^ and NVK.

#### The polymerization initiating ability of the Ir(piq)_2_(tmd)/Ph_2_I^+^/NVK system

**Figure 10:**
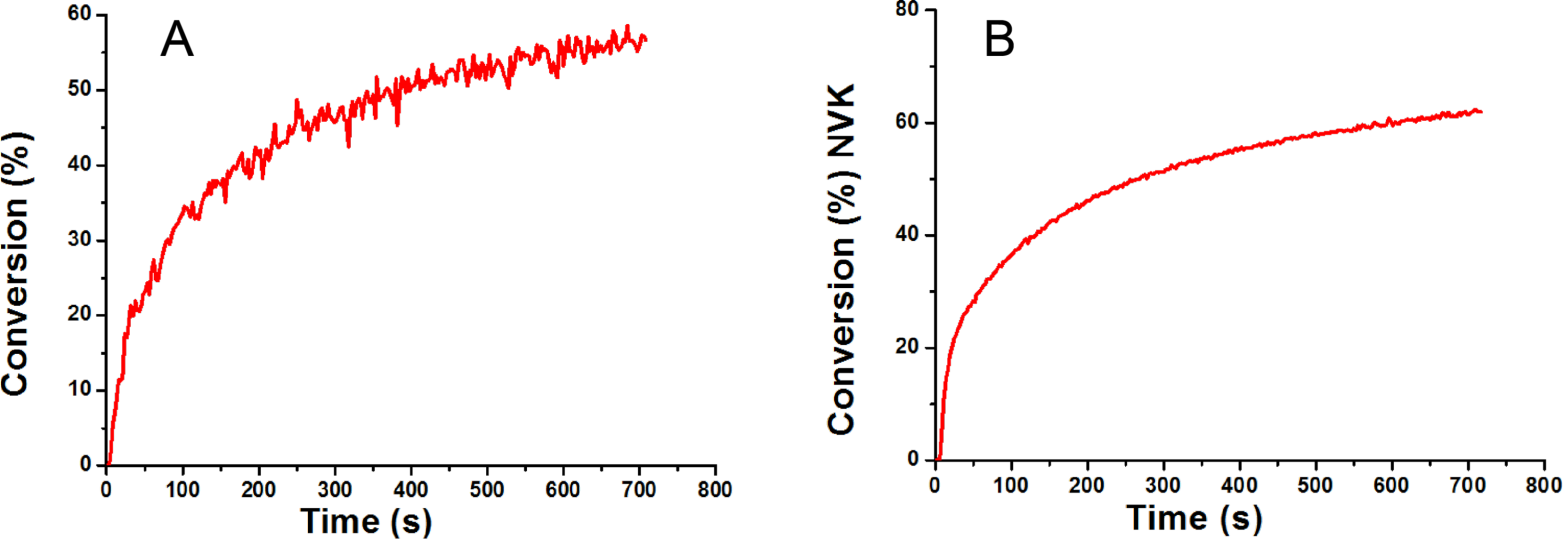
(A) Photopolymerization profile of EPOX; photoinitiating system: Ir(piq)_2_(tmd)/Ph_2_I^+^/NVK (1%/2%/3%); laser diode at 532 nm exposure (~100 mW/cm²); under air. (B) Conversion profile for the NVK double bond in the course of the polymerization.

The polymerization profile for the ring-opening polymerization of EPOX using a Ir(piq)_2_(tmd)/Ph_2_I^+^/NVK initiating system upon a laser diode at 532 nm is depicted in Figure 10A (it was obtained by a classical procedure already presented in [68]). An excellent polymerization is obtained (~60% of conversion for 700 s of irradiation; tack-free coating); on the opposite, in the same conditions, Ir(ppy)_3_/Ph_2_I^+^/NVK leads to a low conversion (<20%) in full agreement with the low absorption of Ir(ppy)_3_ under green lights (see Figure 6). Remarkably, the consumption of the NVK double bonds, followed in the course of the polymerization (see the conversion of NVK in Figure 10B), is in agreement with the mechanism presented in Scheme 8. These results show the interest of developing new PICs with improved or extended light absorption properties.

#### The PIC/MDEA/alkyl halide

**Figure 11:**
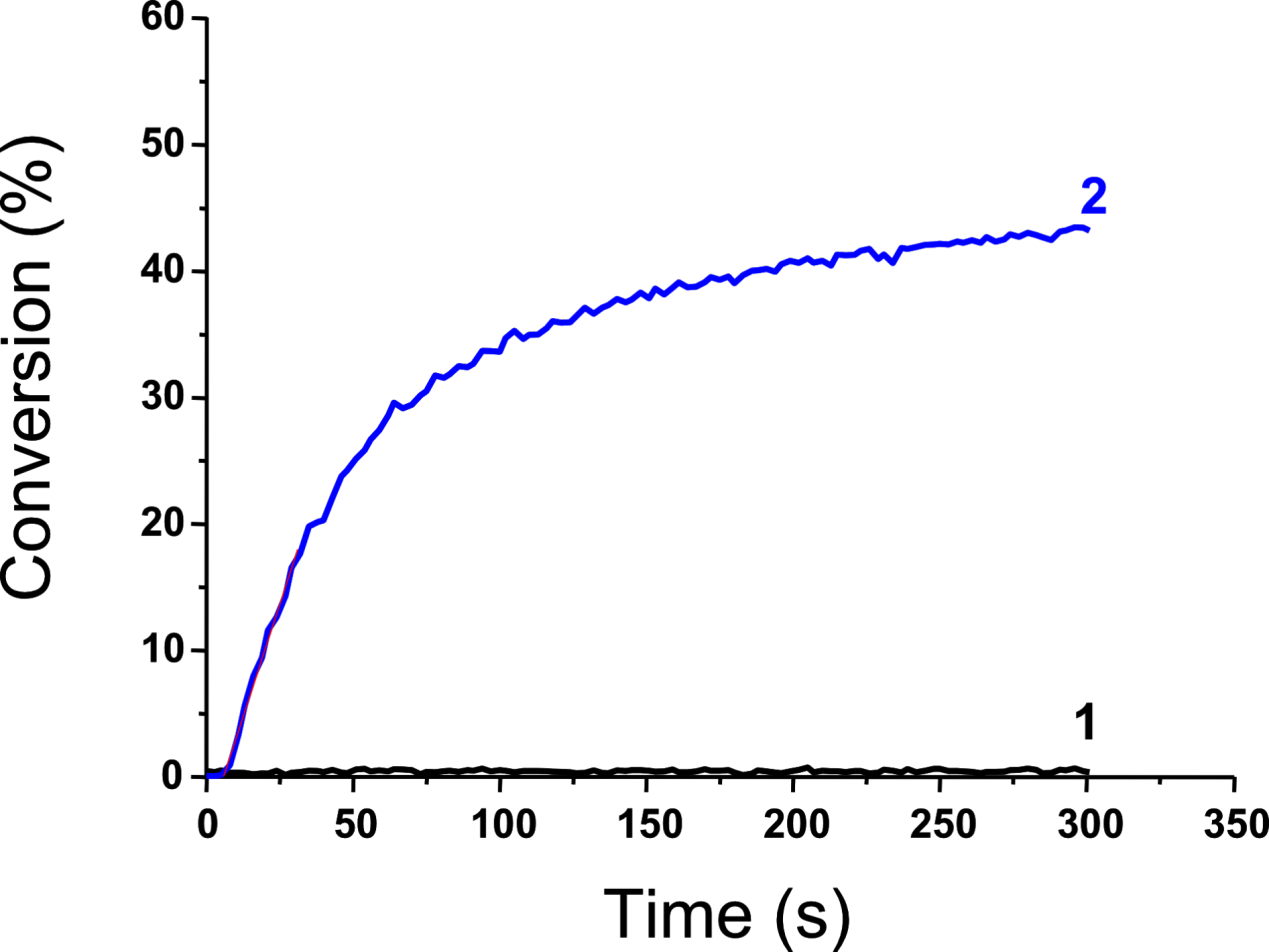
(A) Photopolymerization profile of TMPTA; initiating systems: (1) Ir(piq)_2_(tmd)/MDEA (1%/2%) and (2) Ir(piq)_2_(tmd)/MDEA/R-Br (1%/2%/3%); laser diode at 532 nm; in laminate.

The performance of the new proposed PIC for a reductive cycle in combination with an amine (MDEA) and an alkyl halide (phenacyl bromide; R–Br) to initiate a radical polymerization has been also checked. In full agreement with Scheme 11, the presence of three components is required. Indeed, Ir(piq)_2_(tmd)/MDEA/R–Br is very efficient (Figure 11, curve 2) compared to Ir(piq)_2_(tmd)/MDEA (Figure 11, curve 1).

## Conclusion

Photoinitiator catalysts PICs appear as a new class of initiating systems usable in different photopolymerization reactions: FRP, CP and FRPCP. The associated systems are characterized by an outstanding photosensitivity; the catalytic pathways ensure a regeneration of the PIC and avoid any lost of reactivity upon irradiation. A bleaching of the sensitizer can be observed in excess of oxidation or reduction agent. In this case the polymerization of thick samples can be carried out. With this bleaching, colorless coatings can also be obtained. The search for new PICs and/or new redox agents might be of high interest as depicted by the present proposal of a new PIC (Ir(piq)_2_(tmd)). This allows the design of multicomponent photoinitiating systems based on an excellent Ir complex working at λ > 500 nm, a goal that was never achieved before using other available Ir derivatives (e.g., the well known Ir(ppy)_3_). The development of new catalytic methodologies still remains a huge challenge.
